# Meckel’s Diverticulum Causing Small Bowel Intussusception in Third Trimester Pregnancy, a Case Report

**DOI:** 10.21980/J87H19

**Published:** 2020-01-15

**Authors:** Reece Eric Wilson, Diane Reali-Marini

**Affiliations:** * Kent Hospital Emergency Department, Department of Emergency Medicine, Warwick, RI

## Abstract

Intestinal obstruction complicating pregnancy is a surgical emergency associated with a high incidence of morbidity and mortality for both the mother and fetus. This case report discusses the presentation and treatment of a young female in her third trimester of pregnancy at a repeat visit to the emergency department (ED) with right-sided abdominal pain. She was diagnosed with ileal intussusception secondary to a Meckel’s diverticulum with associated small bowel obstruction (SBO) seen on computerized tomography (CT) imaging. She underwent surgical reduction of the invaginated bowel and diverticulectomy. Making a diagnosis of intestinal intussusception during pregnancy is particularly difficult because common symptoms of intestinal obstruction such as anorexia, nausea, vomiting and abdominal pain are encountered frequently during a normal pregnancy.^1^ Diagnosis is often delayed during pregnancy due to physician hesitancy over the radiation risk to the fetus. While the gold standard for diagnosis is a barium enema, it is often identified on CT scan in adults, and it is regularly managed with surgery.^2^

**Topics:**

Intussusception, small bowel obstruction, pregnancy, Meckel’s diverticulum.

[Fig f1-jetem-5-1-v4]

**Figure f1-jetem-5-1-v4:**
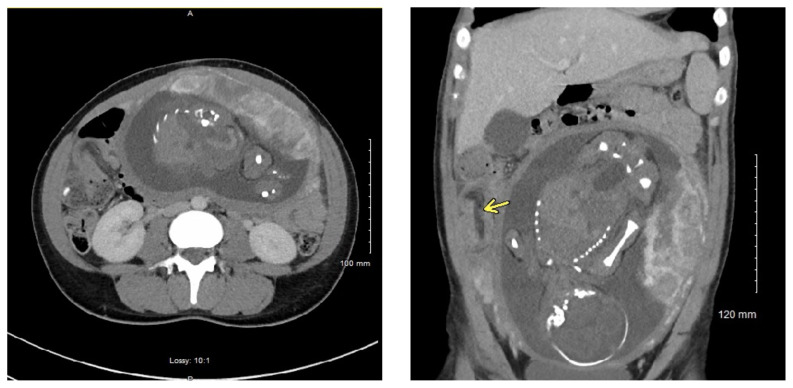
Axial CT Video Link: https://youtu.be/iPkbvEJ1Vms Coronal CT Video Link: https://youtu.be/29GMR59ohnw

## Introduction

Intussusception in pregnancy is a rare but potentially life-threatening surgical emergency. Its associated non-specific symptoms make it challenging to diagnose. Early diagnosis and intervention are essential to reducing the morbidity and mortality to both the mother and fetus.

## Presenting concerns and clinical findings

A 23-year-old G2P1 at 29 weeks female with a past medical history of acid reflux presented to a community ED with intermittent right-sided abdominal pain, nausea, and vomiting for several weeks. She reported daily nausea and vomiting associated with diffuse abdominal cramping after eating. The patient had been seen multiple times at outside hospitals for similar presentation since the onset of her symptoms. Previous evaluations included diagnostic ultrasound imaging of the right upper quadrant to rule out biliary colic, which was unremarkable. Laboratory evaluations included complete blood count (CBC), liver function tests (LFTs) and urinalysis (UA) which were found to be within normal limits. The patient was trialed with home medications for acid reflux with minimal improvement. Vital signs during this encounter were temperature 36.8 ºF, heart rate 84 and regular, respiratory rate 22, blood pressure 144/72 mmHg, and oxygen saturation 98% on room air. Her physical exam was remarkable for a gravid uterus with moderate tenderness to palpation in the right upper and lower quadrants without peritoneal findings.

## Patient Course

Laboratory studies were repeated during the encounter including a CBC, LFTs, UA, basic metabolic panel and were unchanged. A urine protein: creatinine obtained to evaluate for preeclampsia was unremarkable. Magnetic resonance imaging (MRI) of her abdomen was obtained to evaluate for appendicitis given the increasing severity and location of her pain. The MRI showed circumferential thickening of the terminal ileum with adjacent free fluid, but the appendix was not visualized on the MRI. In consultation with the obstetric physician (OB), a CT abdomen and pelvis was ordered for further visualization of the appendix. The CT showed a normal appearing appendix; however, the scan did identify intussusception of the distal small bowel into the cecum with dilation and evidence of SBO. Given the complexity of surgery required, a tertiary facility was recommended by the consulting OB. The patient was transferred and underwent laparotomy performed by a general surgeon and OB team. A Meckel’s diverticulum was found to be the lead point lesion causing the ileal intussusception. The patient’s intussusception was reduced and a diverticulectomy performed. The patient was discharged two days later, and subsequently underwent repeat cesarean section at 36 weeks’ gestation with delivery of a healthy infant.

## Significant findings

A CT scan was obtained which demonstrated distal small bowel intussusception with focal dilation suggestive of a small bowel obstruction in a pregnant female in her third trimester of pregnancy. The fetus can be seen in the uterus. The yellow arrow identifies the area of small bowel intussusception shown by telescoping intestines with associated bowel wall edema.

## Discussion

Intussusception is a rare complication of pregnancy with surgical management being required in most cases. The majority of cases of intussusception are seen in the pediatric population. Intussusception in adults accounts for only 5% of the total number of cases and only 1% of all bowel obstruction cases.^3^ The incidence of intussusception is reported to be 1/30,000 of all hospital admissions, and 1/1300 of all abdominal operations.^4^ In over 80% of cases there is an attributable underlying cause, such as a tumor, polyp, or Meckel’s diverticulum as in this case.^5^ Between 8% and 20% of the total cases in adults are idiopathic without identifiable source. Approximately twenty-five percent of small bowel intussusceptions are caused by malignant lesions.^6^

Making a diagnosis of intestinal intussusception during pregnancy is particularly difficult because common symptoms of intestinal obstruction such as anorexia, nausea, vomiting and abdominal pain are encountered frequently during a normal pregnancy.^1^ Additionally complicating this case, the patient had a long-standing history of acid reflux. During previous ED encounters, her symptoms were attributed to worsening of this condition which delayed her diagnosis. The abdominal exam is challenging in second and third trimester pregnant patients secondary to the growing uterus. Physical findings of guarding and rigidity may not be elicited in the third trimester of pregnancy due to stretched abdominal muscles.^8^

While contrast enema remains the gold standard for the diagnosis of intussusception, CT imaging is now widely used in patients with undifferentiated abdominal pain. As a result, it is often the imaging modality with which intussusception is diagnosed. It may provide important additional information including cancerous lesions, lymphadenopathy, free fluid and proximal bowel dilation.^9^ Computerized tomography has a reported diagnostic accuracy of 58%–100%,^5^ while abdominal ultrasound (US) is 60% accurate in adults. Plain radiography offers little clinical utility.^10^ Ultrasound is utilized frequently for the diagnosis of pediatric intussusception. The distorted anatomy that occurs in pregnancy from the gravid uterus makes the utility of ultrasound limited in these cases. Treating clinicians are cautious in the use of CT scans because of possible teratogenicity. As exemplified in this case, an MRI was initially obtained to evaluate for appendicitis in an attempt to limit radiation exposure to the fetus. A single-pass CT scan of the abdomen and pelvis with intravenous and oral contrast can potentially expose the fetus to 25mGy (milli-gray) of radiation. This exposure puts the fetus at a 1% increased risk of developing childhood cancer which must be weighed against the benefit of possibly diagnosing a surgical issue with the mother.^11^ There is limited information regarding diagnostic accuracy of MRI in intussusception but the radiographic findings of bowel wall edema and telescoping bowel are similar to that of CT.^12^

Once the diagnosis of intussusception has been made, surgical intervention is almost always necessary. While pediatric intussusception usually can be reduced with an enema (barium, air, saline), most adult cases require surgery.^4^

In conclusion, patients with intussusception during pregnancy present with very non-specific symptoms and unreliable physical exam findings. The combination of these findings along with the rarity of this presentation in adults makes intussusception a clinically difficult diagnosis. Clinicians must maintain a high suspicion for this disease process because early surgical intervention is key to reducing morbidity and mortality for both the mother and the fetus.

## Supplementary Information










