# Supplementary figures and images for: Meckel’s Diverticulum Causing Small Bowel Intussusception in Third Trimester Pregnancy, a Case Report
Source: J Educ Teach Emerg Med. 2020 Jan 15;5(1):V4–7. doi: 10.21980/J87H19 (PMC10332542; doi:10.21980/J87H19)

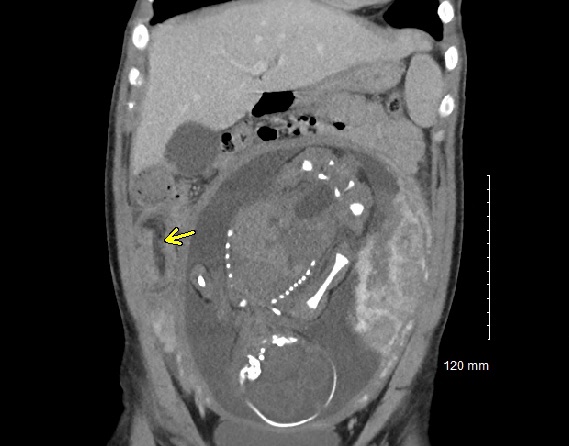

Supplement: Supplementary file 1 [file jetem-5-1-v4-supp1.jpg]

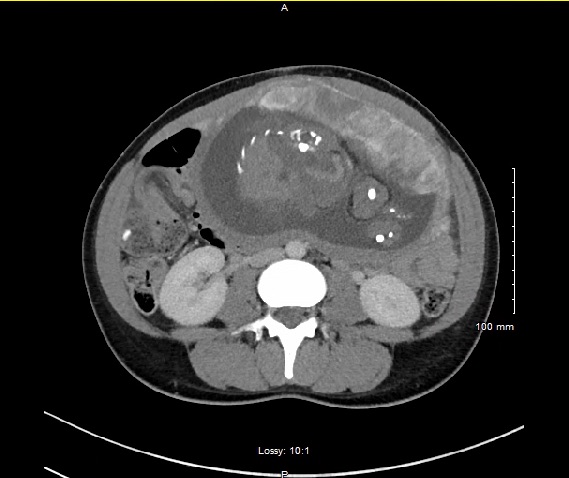

Supplement: Supplementary file 3 [file jetem-5-1-v4-supp3.jpg]

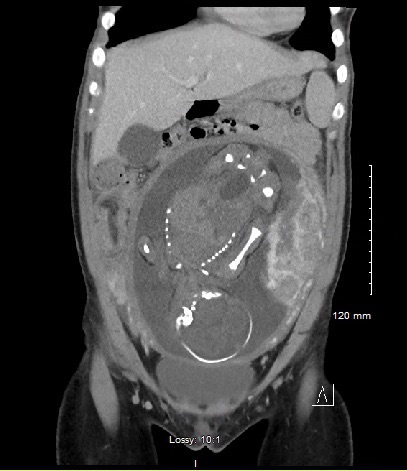

Supplement: Supplementary file 5 [file jetem-5-1-v4-supp5.jpg]
